# Potentiometric Electronic Tongues for Foodstuff and Biosample Recognition—An Overview

**DOI:** 10.3390/s110504688

**Published:** 2011-04-28

**Authors:** Patrycja Ciosek, Wojciech Wróblewski

**Affiliations:** Department of Microbioanalytics, Warsaw University of Technology, Noakowskiego 3, 00-664 Warsaw, Poland; E-Mail: wuwu@ch.pw.edu.pl

**Keywords:** ion selective electrodes, electronic tongue, principal components analysis, artificial neural networks, partial least squares, sensor array

## Abstract

Potentiometric sensors are attractive tools for the fabrication of various electronic tongues that can be used in wide area of applications, ranging from foodstuff recognition to environmental monitoring and medical diagnostics. Their main advantages are the ability to modify their selectivity (including cross-sensitivity effects) and the possibility of miniaturization using appropriate construction methods for the transducer part (e.g., with the use of solid-state technology). In this overview various examples of the design, performance, and applications of potentiometric electronic tongues are presented. The results summarize recent research in the field conducted in the Department of Microbioanalytics, Warsaw University of Technology (WUT).

## 1. Introduction

Electronic Tongues (ETs) are systems composed of a set of sensors (a sensor array) and a data analysis system (Pattern Recognition block, PARC), which allows one to extract useful information from sensor responses [1–7]. Such systems are dedicated to the automatic, fast, qualitative and/or quantitive analysis of samples with complex matrices. Their task is usually to recognize characteristic properties such as taste, origin or nutritional value, to estimate the content of key components, to detect abnormalities, *etc.*

A wide variety of chemical sensors can be employed in the design of electronic tongues: electrochemical (voltammetric [8–11], potentiometric [6,12], impedimetric [13,14]), optical [15] or enzymatic sensors (biosensors, [16]). However, most of these systems are based on potentiometric sensors, especially Ion-Selective Electrodes (ISEs). All commercially available systems, *i.e.*, the Taste Sensing System from Anritsu Corp., Atsugi (Japan), ASTREE from Alpha MOS (France), the Multiarray chemical sensor from McScience (Korea), are based on potentiometric measurements [1]. Beside ISEs, nowadays ISFETs are also gaining bigger interest as sensors forming ET sensor arrays—they are used in ASTREE, as well as in other devices [17].

The main disadvantages of potentiometric measurements are their temperature dependence, the influence of solution changes, and adsorption of solution components on membrane surfaces, that can affect the nature of the charge transfer. The effect of those factors can be minimized by controlling the temperature, washing of the electrodes with solvents limiting the adsorption, application of anti-fouling layers, *etc.* The advantages of ISEs, *i.e.*, their well-known principle of operation, low cost, easy fabrication, simple set-up, the possibility of selectivity modification to detect many various species, and their close similarity to the natural mechanism of molecular recognition—have made them the most popular in the development of sensor arrays for ET applications.

In this overview, we summarize the research conducted in the Department of Microbioanalytics at Warsaw University of Technology, aimed at the development of potentiometric electronic tongues. First, various architectures of potentiometric sensor arrays are presented, then data analysis procedures that were used in a variety of analysis, and finally a few examples of analytical problems solved with the use of ET systems are discussed.

## 2. Sensor Arrays Based on Various Architectures

**Figure 1.**
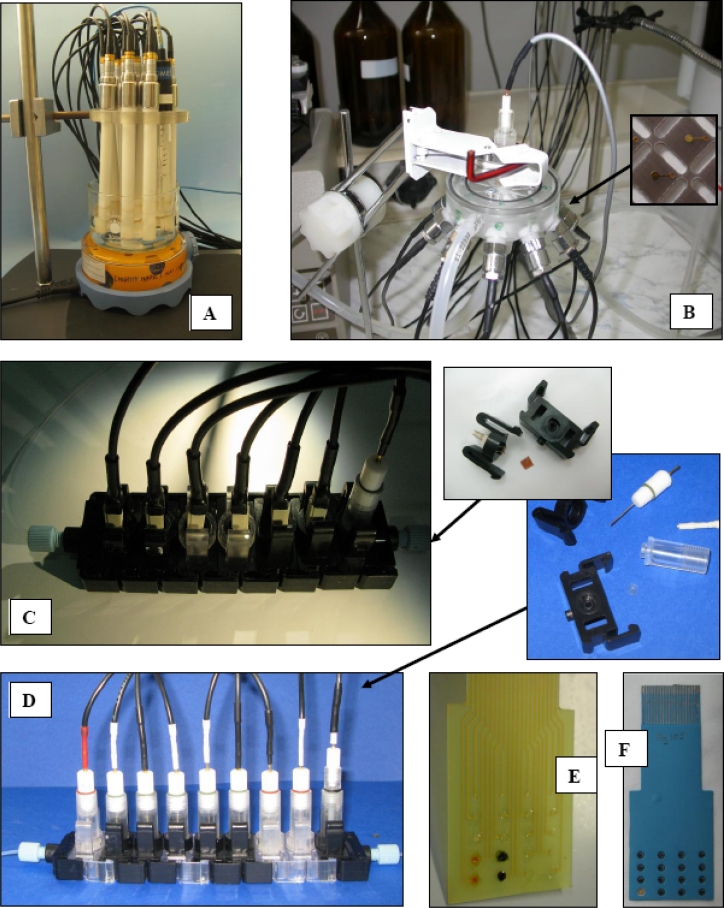
Sensor arrays composed of potentiometric electrodes (**A**) ISEs in Philips bodies (**B**) flow-through system with circular measurement cell and miniaturized SSEs (**C**), (**D**) linear flow-through systems with (**C**) SSEs (**D**) miniaturized ISEs with classical architecture (**E**), (**F**) integrated microelectrode arrays based on (**E**) epoxy-glass laminate (**F**) LTCC.

Most early models of electronic tongue systems were based on sensors with classical architectures. Potentiometric, as well as voltammetric ETs were built as sets of electrodes in Philips bodies or sets of disc electrodes. The research on sensor arrays in the Department of Microbioanalytics, WUT, also started with the use of classical Ion-Selective Electrodes, however, in contrast to the usually applied cross-sensitivity approach, selective sensors were used. Such sensor arrays [Figure 1(A)] combined with pattern recognition tools, were applied to the qualitative analysis of mineral waters and apple juices [18]. The procedure of quantitative evaluation of a particular sensor to discriminate between different classes of samples was proposed (F-factor). Electrodes characterized by higher F-factors were chosen to form the final sensor arrays, whose classification capabilities were enhanced—after reducing of number of the sensors, and the device was capable of reliable discrimination between different brands of mineral waters and apple juices [18].

The same sensor array architecture was applied to the qualitative analysis of various brands of orange juice, tonic, and milk. A new approach was proposed for such tasks—the fusion of two types of sensors: classical selective and partially selective ones in one versatile array [19]. The processing of the sensor array’s response by means of Principal Component Analysis (PCA) and Back Propagation Neural Networks (BPNN) allowed the discrimination between different brands of various beverages with very high accuracy [20].

The presented systems based on classical ISEs were used in stationary measurements. For monitoring of foodstuff production, flow-through analysis can be more advantageous, thanks to the resulting shortening of response time, convenience of calibration and possibility of miniaturization. Moreover, in such dedicated flow systems, miniaturized transducers can be applied, which demand less chemicals and smaller sample volumes, leading to significant reductions of the analysis costs. Miniaturized electronic tongues can be also coupled with various miniaturized pretreatment systems, if the samples to be analyzed need any special preparation (e.g., filtering, dilution, *etc.*). Recently, electronic tongues working as flow injection- and sequential injection analysis systems have been used in a variety of applications which were summarized in a review by M. del Valle *et al.* [21].

Our first works on flow-through electronic tongues were based on the use of miniaturized solid-state potentiometric sensors [22] and circular flow-through cells [Figure 1(B)] [23–25]. A simple technique, *i.e.*, membrane solution casting on the surface of the planar Au transducers, was applied for the preparation of classical ion-selective and partially selective microelectrodes. The performance of the designed system was again tested in the qualitative analysis of various brands of beverages such as beer, juice and milk. The sensor array responses were processed by BPNN or Partial Least Squares—Discriminant Analysis (PLS-DA), which lead to high classification results for the set of testing samples [19,23,24].

The circular flow-through cell was however equipped with classical flow channels, which were suitable for foodstuff monitoring, where the volume of the sample is not a crucial issue. According to large number of samples needed to calibrate ET systems, it would be desirable to reduce sample volume. There is a need to develop flow-through systems with minimized flow and consumption of the sample on the order of few milliliters. A modular flow-cell system [26] has been developed by MEDBRYT (Warsaw), in cooperation with our group, within the framework of the FP6 WARMER project. The main element of this system is a sensor housing, presented in Figure 1(C). It is provided with two arms, enabling horizontal connection with other housings—or with flow-input/output pieces. A set of modules can be connected into a measurement loop-flow [27]. Each element of the system can be connected and disconnected quickly and easily, allowing us to construct various sensor arrays dedicated to specific types of analysis. Such a sensor array architecture equipped with Solid-State Electrodes (SSEs) was used for the analysis of samples obtained during methane fermentation (anaerobic digestion) of whey in a sequencing batch bioreactor. The investigated samples, measured in modular flow-cell system, were discerned according to their Volatile Fatty Acid (VFA) content and Chemical Oxygen Demand (COD) [28]. In this way ET was used as a tool capable of estimation of the stage of fermentation and stability of the process, which can be thrown out of balance by some of the compounds present in culture medium.

The modular flow-through cell was dedicated for various planar sensors (mainly for SSEs [22,27]). Due to some disadvantages of planar sensors, e.g., the necessity of the formation of solid contact, a new miniaturized ion-selective electrode design compatible with the flow-through array was proposed [29]. Such an electrode has a classical architecture, in which ion-selective membrane is in contact with the inner electrolyte that is placed in an electrode body and the transducer part is based on a Ag/AgCl electrode connected to a multiplexer that passes the amplified signal to a computer [Figure 1(D)]. The system was applied to the monitoring of periodic anaerobic digestion [29]. The applied flow-through array allowed on-line process control, and regarding future applications, its modular architecture is advantageous when assembling a sensor set.

Miniaturization of single sensors is one of two main strategies towards miniaturization of sensor arrays in ET systems. The second is the development of integrated sensor arrays, fabricated on a single substrate [2,30,31]. In our works, we proposed 16- or 20-electrode sensor arrays for ET applications [32]. The integrated microelectrode arrays were fabricated on the basis of two materials: epoxy-glass laminate [Figure 1(E)] and Low Temperature Cofired Ceramics (LTCC) support [Figure 1(F)]. Their architecture, dimensions, and multilayer structure are similar. Various polymeric membranes based on poly(vinyl chloride) (PVC) or polyurethane (PU) formed the chemosensitive polymeric layers of the potentiometric sensors of the arrays [33].

The performance of the integrated system based on epoxy-glass laminate and PVC membranes was checked in the recognition of various juice brands. PLS was used as a classifier, and the results of the classification were very satisfactory—all the samples from the training set and the testing set, were correctly recognized [34]. In another example, the same device was used for the classification of milk originating from various producers. The system was capable of recognition of such milk samples with high correctness. Moreover, the introduction of miniaturized reference electrode on the same substrate, also provided satisfactory results, which could be helpful in future construction of hand-held electronic tongue systems [35]. Different types of polymeric materials, *i.e.*, PU doped with various electroactive components, were applied in integrated ETs on epoxy-glass laminate for the recognition of tea and herbal products. The responses of the prepared integrated sensor array were analyzed with the use of PCA and Principal Components Regression (PCR), in order to differentiate between various types of beverages (black tea/red tea/green tea/herbals) and to discriminate among various kinds of them [36]. Integrated sensor arrays were also fabricated on a ceramic support, with the use of LTCC technology. The array of microelectrodes covered with polymeric layers of various selectivities was applied as an electronic tongue to differentiate between various diet supplements [37] and for monitoring of cell cultures in microbioreactors [38]. The presented integrated arrays, based on epoxy-glass laminate support as well as LTCC, with microelectrodes exhibiting high and partial selectivity, can be used in portable electronic tongue systems and for the analysis of small volume samples.

## 3. Data Analysis for Potentiometric Sensor Arrays

**Figure 2.**
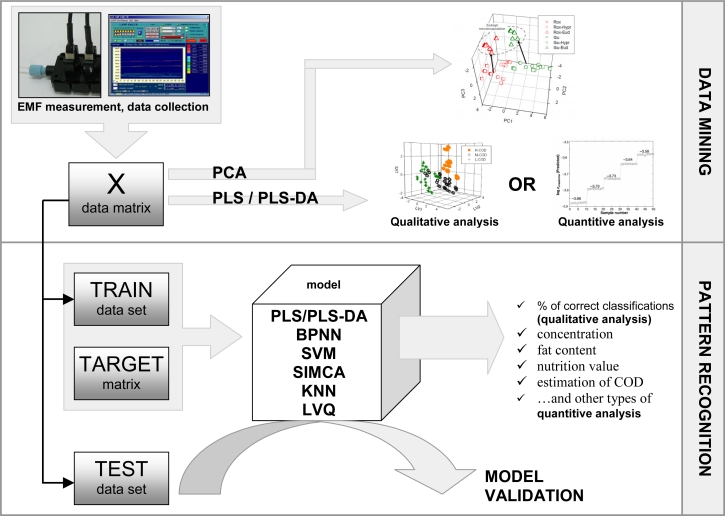
Data analysis procedures for potentiometric sensor arrays.

Sensor array measurements produce large amounts of data that must be analyzed with the use of various numerical techniques. If the classes of the samples are unknown, or basic differentiation ability of the sensors is checked, unsupervised data mining methods are used. The most popular procedures, that allow one to visualize clustering of chemical images of similar samples, are PCA and PLS-DA for qualitative recognition [18,29] and PLS for quantitative analysis [39] (Figure 2).

The main task for ET system is the recognition of unknown samples and the estimation of their characteristic parameters. Therefore two stage data analysis based on pattern recognition methods for supervised learning are usually performed. First, the data matrix is divided into two sets: training set and testing set. Then, a model is built by tuning the parameters of the procedure in order to produce appropriate target vectors in response to sensor signals obtained for the training set. Then the testing set is applied to validate the system (Figure 2), and final classification ability (in qualitative analysis) or error of concentration, fat content, nutrition value, COD, *etc.* (in quantitative analysis), is estimated.

So far, many pattern analysis techniques for data analysis of sensor array measurements were presented [1]. The most popular are: PCA, PLS, PLS-DA, PCR, Artificial Neural Networks (ANN), and Soft Independent Modeling of Class Analogy (SIMCA) [2,4,5]. There is no single, optimal procedure to analyze sensor array data, giving both reliable and low level of error results, which can be applied in any classification problem. Some general remarks on strategy of choosing a most appropriate data treatment method can be found in [3,4,6]. Comparisons of various pattern recognition methods for electronic tongue systems were presented in [40] and [41]. An ET was applied to the qualitative analysis of various brands of milk. The classification results obtained for five PARC tools were compared. The most straightforward method—K-Nearest Neighbors (KNN) was used as a reference for other classifiers, because of its high accuracy and no necessity of training. Other PARC techniques, *i.e.*, PLS and Soft Independent Modeling of Class Analogy (SIMCA) also demonstrated satisfactory results. The general rule of reliability and accuracy of neural networks for the analysis of sensor array responses was confirmed, which is linked with their nonlinear functioning and the capability to model the overlapping of classes of the measured data. Although BPNN are the common type of neural networks used in ET systems, we found that Learning Vector Quantization (LVQ) nets [41] and Support Vector Machines (SVM) [25] demonstrated even better performance.

A novel strategy of data analysis for ET system was presented in [42]. It was proved that the use of a supervised method also in the feature extraction phase enhanced the fruit juice classification capability of a sensor array fabricated with classical ISEs. Comparison of direct processing with the use of BPNN, raw data processed by PLS-DA, and two-stage processing (PCA outputs processed by BPNN, PLS-DA outputs processed by BPNN) showed that a considerable increase of classification capability occurred in the case of the new method. Fusion of linear and nonlinear processing closely simulates the natural signal processing and the application of a classifier combination helps to discern various classes of samples in more complicated regions of pattern space.

Signal stability of SSEs can be insufficient for some ET applications, when very long measurement times are necessary. However, in such cases, various technical and chemometric procedures have been proposed in the literature to solve this problem (e.g., correction of the baseline, drift modeling, the use of recalibration solutions). A simple method to overcome the signal instability of developed SSEs (integrated on a ceramic support) was proposed in our group and presented in [37]. The eventual sensor drift was effectively compensated using the signal of the sensor array measured in a reference solution.

## 4. New Challenges for Applications of Potentiometric ETs

In contrast to their natural counterparts, ET systems are usually adapted to specific tasks. The most evident application is the automatic quality control in the foodstuffs industry, however during the last few years many more were proposed: environmental and industrial analysis (monitoring of water contamination, detection of chemical weapons, drugs…), non-invasive medical diagnostics (analysis of human saliva, urine, sweat…), the detection of toxigenic fungi, other contaminations in feed, monitoring of bacterial growth, *etc.* [1–8].

**Figure 3.**
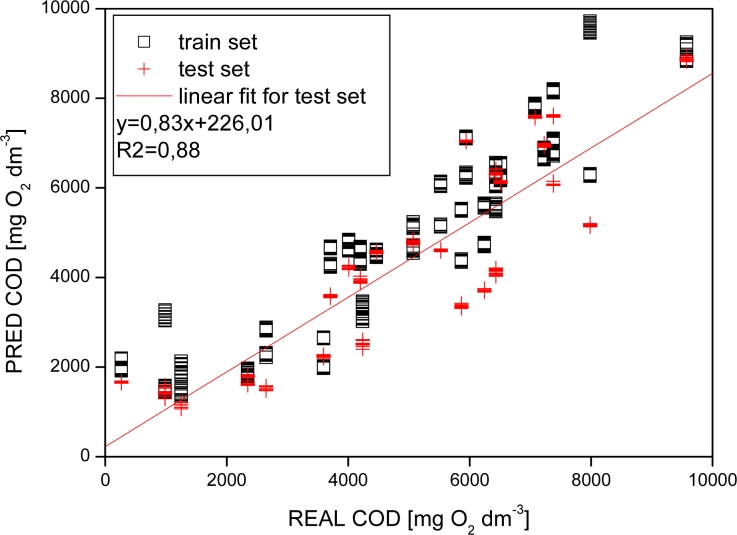
PLS prediction of Chemical Oxygen Demand (COD) in methane fermentation media. The prediction was based on the analysis of sensor array responses obtained from modular flow-through cell ET (Copyright Elsevier B.V.) [29].

In our works, the presented ET systems were mainly used for the classification of various beverages [18–20,23–25,34,35,40–42], however we also proposed some new applications, e.g., the monitoring of methane fermentation [28,29,43]. In the last few years ETs were applied for various fermentation monitoring, such as *Aspergillus niger* fermentation, light cheese production, batch *Escherichia coli* fermentation, as systems capable of fast, inexpensive, automated and on-line control of the process [7]. As pointed out in [7], till now only a few attempts were made to apply sensor arrays, both electronic noses and tongues, to the monitoring of biogas production. The main reason is the very complex and poorly reproducible composition of process media in comparison to other fermentations. In our works a modular flow-through array of SSEs [28] and a modular flow-through array of miniaturized ISEs of classical architecture [29] were used for the analysis of samples obtained during methane fermentation (anaerobic digestion) of whey in sequencing batch and periodic bioreactor. The samples were classified according to their COD and VFA content in liquid phase of process media, in order to estimate the stage of fermentation process. Exemplary results of COD determination are presented in Figure 3.

**Figure 4.**
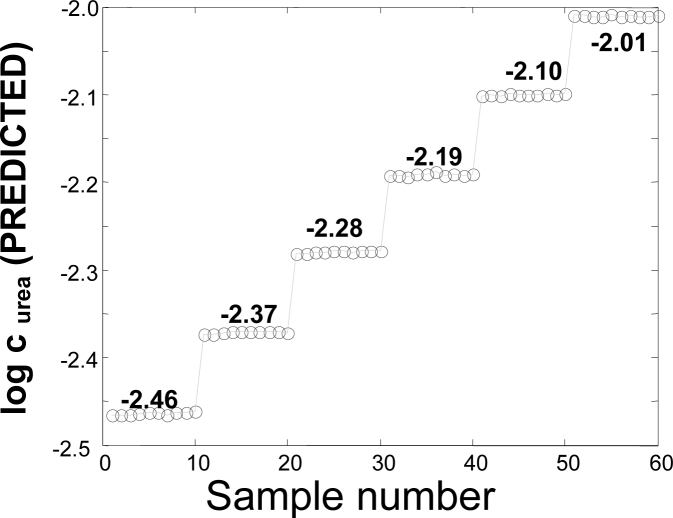
Predicted responses of bioelectronic tongue in the case of dialysis fluids containing various amounts of urea (With kind permission from Springer Science+Business Media: Microchim) [39].

A bioelectronic tongue composed of classical ISEs and biosensors was used in non-invasive monitoring of dialysis treatment [39]. The measurements of dialysate fluids containing creatinine and urea additives at concentrations commonly present in post-dialysate fluids were conducted and the responses of sensors were processed using PLS analysis. The device was capable of determining urea and creatinine with satisfactory correctness (Figure 4). Moreover, simulated samples typical of the initial and final phases of dialysis could be differentiated.

**Figure 5.**
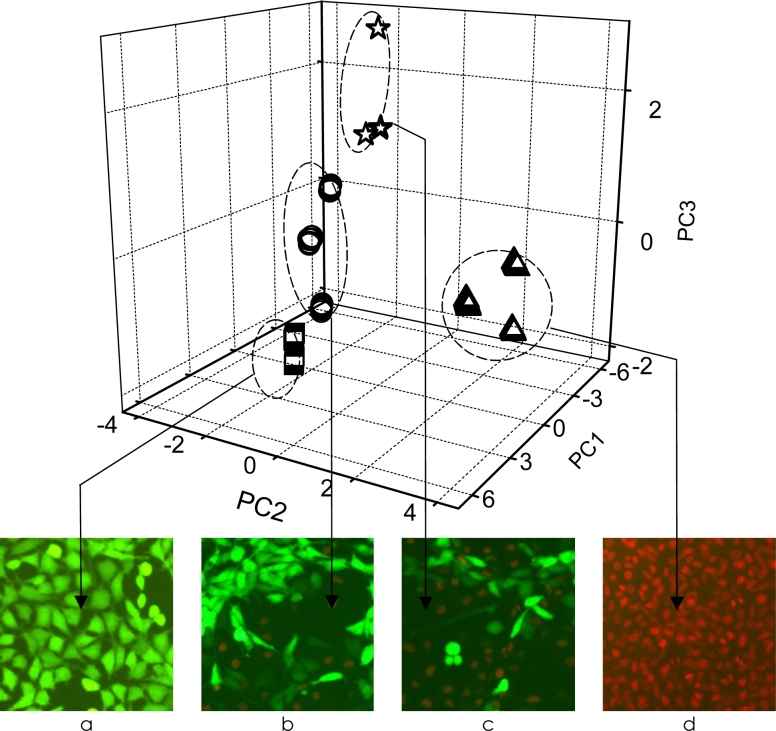
PCA plot of the toxic effects of 1,4-dioxane on Vero cells: (**a**) Vero cells cultured in a routine way, (**b, c, d**) Vero cells treated with different concentrations of 1,4-dioxane. The application of PI (marker of dead cells) and FDB (marker of living cells) showed nearly 100% (a), 70% (b), 40% (c) and 0% (d) of living cells (With kind permission from Springer Science+Business Media: Analytical and Bioanalytical Chemistry) [38].

Monitoring of cell cultures in microbioreactors is a crucial task in cell bioassays and toxicological tests. For such purpose we proposed a miniaturized sensor array fabricated using LTCC technology [38]. The developed device was applied to the monitoring of cell-culture media change, detection of the growth of various species, and in toxicological studies performed with the use of cells [38]. A PCA plot of the toxic effects of 1,4-dioxane on Vero cells is presented in Figure 5. Noninvasive monitoring performed with the LTCC microelectrode array can be applied for future cell-engineering purposes.

Some properties of food products, such as nutritive value and consumption usability are hard to investigate by classical analytical techniques. We presented the first attempts toward the estimation of quality of edible plants with a sensor array system in [44].

**Figure 6.**
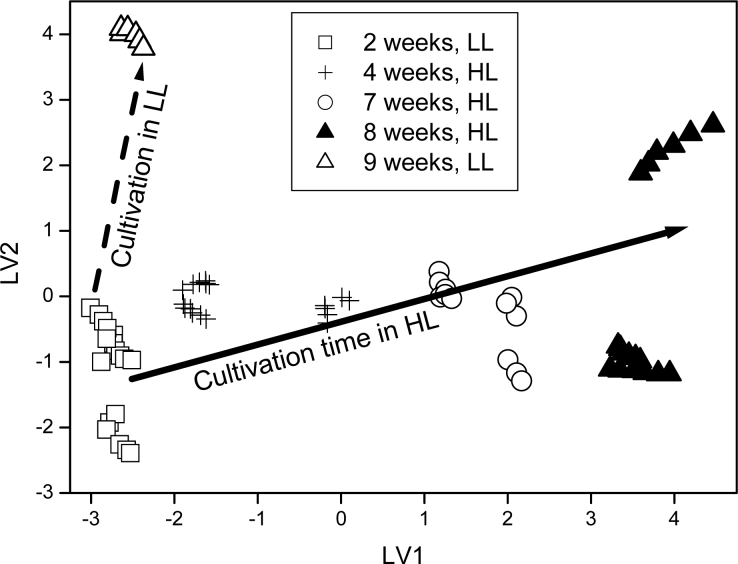
PLS plot of guinea grass samples cultivated for various time in high light (HL) and low light (LL) conditions (Copyright Wiley-VCH Verlag GmbH & Co. KGaA. Reproduced with permission) [44].

An electronic tongue based on an array of ISEs was applied to the study of the type of plant metabolism (distinguishing between two types of cereals), and to recognize the cultivation time and light intensity during growth. Those factors influence the development of edible plants and thus affect their quality as food components. High ability of the electronic tongue system to distinguish among various subtypes of C4 plants, plants cultivated in various light conditions and for different periods of time was observed (exemplary results are presented in Figure 6), and thus usability of ET systems to the monitoring of crops and estimation of the quality of plants was proved [44].

**Figure 7.**
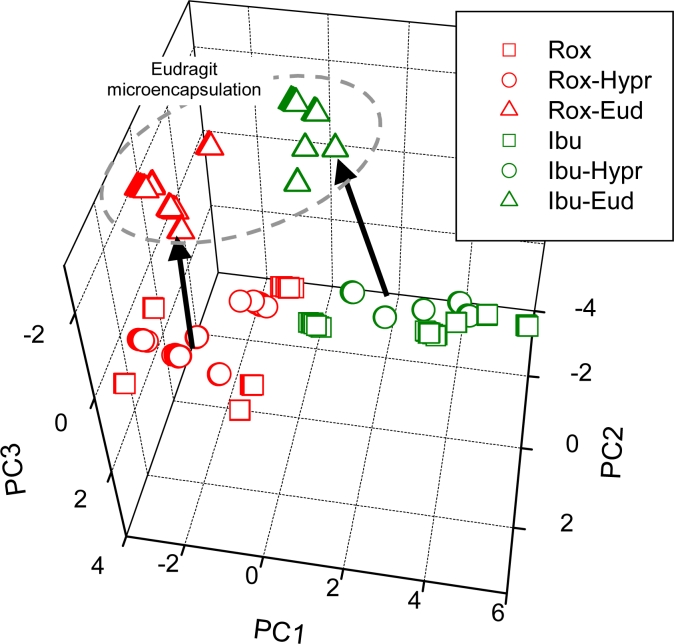
PCA plot of unmodified and modified API samples–eudragit modification changes chemical images of API in the same direction [45]. Ibu–pure ibuprofen; Ibu-Hypr–ibuprofen modified by hypromellose; Ibu-Eud–ibuprofen modified by eudragit; Rox–pure roxithromycin; Rox-Hypr–roxithromycin modified by hypromellose; Rox-Eud–roxithromycin modified by eudragit (Copyright Elsevier B.V.) [45].

Potentiometric ETs can produce chemical images of samples, whose changes can be correlated with taste sensations. In [45] we presented a sensor array coupled with PCA for the detection of effect of microencapsulation of two Active Pharmaceutical Ingredients (APIs)—ibuprofen and roxithromycin—which influenced their taste properties. PCA allowed one to distinguish medicine samples by the presence of changing-taste substance criterion/standard, *i.e.*, chemical images obtained from the measurements of pure API solutions and API encapsulated with taste-masking additives (eudragit and hypromellose) were significantly different. Moreover, the character of change obtained thanks to microencapsulation was the same in both APIs, proving, that the “sensed taste” becomes similar in both formulations after eudragit modification (Figure 7). The obtained results showed that potentiometric ET can be used for analysis of masking effects in drugs and detection of encapsulation effect [45].

## 5. Summary

Recently, many research works have been undertaken in the field of electronic tongue systems and many different samples were analyzed with such systems. The advantages of ETs surpassing the natural sense of taste can be noticed: ETs are not subjective, they are adaptive and they do not become tired or infected, things which can always influence human panellists. Moreover, with the use of such systems, a whole range of samples can be analyzed (including nonedible and toxic samples), and not detectable by their natural counterparts substances can be detected.

Potentiometric electrodes are the most widely used sensors in electronic tongue systems. This review summarizes the research that was conducted in the Department of Microbioanalytics at Warsaw University of Technology, that aimed at the development of potentiometric electronic tongues. Various potentiometric sensor array architectures, new propositions of procedures for analysis of potentiometric array responses, and new, non-typical applications of potentiometric ETs are presented. Many possible applications and many advantages of ET systems, make them an interesting research topic whose further development in the coming years is expected.
